# Commentary: Development of Defensive Actions in Small-Sided and Conditioned Games With Offensive Purposes in Futsal

**DOI:** 10.3389/fpsyg.2021.764995

**Published:** 2021-11-11

**Authors:** Italo Sannicandro, Gaetano Raiola

**Affiliations:** ^1^Department of Humanities, Literature, Cultural Heritage, and Education Sciences, University of Foggia, Foggia, Italy; ^2^Human Sciences Department, University of Salerno, Salerno, Italy

**Keywords:** ecological dynamic approach, small-sided games, soccer, futsala, youth sport

## Introduction

Some of the topics covered in a recently published article by D. Pizarro et al. (2020): “Development of Defensive Actions in Small-Sided and Conditioned Games with Offensive Purposes in Futsal,” *Front. Psychol*. 11:591572, have caught our attention.

We wish to congratulate the authors for their successful and original study and to offer some comments on the issues covered with the goal of highlighting some related aspects.

To prepare athletes for the physiological demands of soccer and futsala competitions, coaches need to identify effective training modalities. Small-sided games (SSGs) are frequently used by coaches to develop the technical abilities of players as well as their conditioning capacity through the recruitment of sport-specific movement patterns.

SSGs comprise a very wide variety of exercises that can be manipulated by the staff according to the goal of a single session or the week of training. SSGs are employed in the training programs of both professional soccer players and young soccer players.

The authors duly described the advantages derived from the use of SSGs for learning defensive tactical skills in young futsala athletes, and rightly highlighted the advantages derived from a non-linear pedagogy based on the ecological dynamics approach (EDA) to skill acquisition.

## Small-Sided Games and Youth Training

The relationship between the modality of organizing a youth training approach and the effects of this approach affect the effectiveness of skill learning as well as issues relating to injury risk in young athletes and to health (Renshaw et al., 2019).

With regard to learning effectiveness, for many years, the analytical approach to setting motor task demands prevailed over other modes of organizing motor and technical tasks. This reflected the widely held belief in the effectiveness of linear motion patterns, which played a dominant role in the motor behavior theories developed from the 1960's through to the 2000's, and which continue to prevail in current practice.

Indeed, the traditional technical method to teaching soccer skills has remained predominant to date. Through this approach, the coach plans a sequence of prescriptive exercises, based on simulations of a part of the game, and these exercises are performed under the direct command of the coach.

The rationale behind this approach assumes that a certain degree of skill must first be acquired before being put into effect in the context of a match; however, it is also true that the demands of mastering a soccer game require much more than just physical or technical skill (Barba-Martín et al., 2020).

In fact, in open skill sports perceptual, decisional and cognitive aspects play a very significant role in solving motor problems. In this regard, the relationships between motor and cognitive functions have been highlighted by neuroimaging studies, which provided evidence suggesting that motor and cognitive processes draw on common neural mechanisms and resources (Stuhr et al., 2018). This interesting review by Stuhr et al., conducted on populations of teenagers, reported that the relationship between motor skill proficiency and cognitive performance is task-specific. This means that the structuring of training sessions for young team-sport athletes must reproduce the same motor problems that the young person will face during the competition.

With regard to injury risk in young soccer players, the current debate on “early specialization vs. diversification” in youth sport has meant that technical staff must embrace considerations that not only increase the effectiveness of methodological approaches but also reduce injury risk (van der Fels et al., 2015). In fact, the EDA specifically enables young players to select the movement pattern he considers to be the most functional for solving a motor problem. And this can also extend to movement patterns that are not sport-specific.

In this regard, Pizarro et al. underline the fact that the EDA applied through SSGs promotes “effective motor exploration” that facilitates the emergence of functional movement solutions within game situations.

This effective motor exploration is crucial for the training process of young soccer players because it generates a relationship between learning from the motor exercise and learning the environment within which the exercise must be performed.

Coaches can gain significant benefits by structuring exercises using the ecological dynamic approach: indeed, motor exploration is exactly what the young athlete is required to do during a game, where all the actions entail repeating (skills) without repetition in a context of continuous problem solving.

Conversely, the traditional approach to learning technical and tactical skills has always been analytical. It typically involves a very high number of repetitions and a training regime divided into “blocks,” and of consequence the overloading of certain muscle and joint structures in young players (Jayanthi et al., 2020).

## Discussion

Some considerations must be made regarding the use of SSGs based on an EDA. Unlike the traditional approach which provides for the precise quantification of load (with the number of sets and repetitions being decided in advance), the use of SSGs means that the types and number of times each movement is performed by during the exercise is not known beforehand. Similarly, the level of intensity at which the exercise is performed and the load of the internal and external parameters cannot be predicted.

A number of useful tools are available today for monitoring external and internal load (e.g., GPS and heart rate monitors), but these tools measure their parameters during the execution of the exercises; in fact, some tools only provide the results at the end of the exercise. Ultimately, the monitoring of load takes place both during and after the execution of an exercise, and it is most useful for planning future training sessions rather than for previewing the intensity of the session underway.

In conclusion, SSGs offer obvious advantages in relation to the learning of technical and tactical skills in football and futsala. They also limit the risk of injury in young players caused by the excessive repetition of set movement patterns, but their format makes it difficult to manage exercise intensity.

A more balanced organization of training sessions for young players which combines a sport-specific element with a general training phase is probably the best option to ensure that the technical objectives are reached whilst safeguarding the health of young athletes.

## Author Contributions

All authors listed have made a substantial, direct and intellectual contribution to the work, and approved it for publication.

## Conflict of Interest

The authors declare that the research was conducted in the absence of any commercial or financial relationships that could be construed as a potential conflict of interest.

## Publisher's Note

All claims expressed in this article are solely those of the authors and do not necessarily represent those of their affiliated organizations, or those of the publisher, the editors and the reviewers. Any product that may be evaluated in this article, or claim that may be made by its manufacturer, is not guaranteed or endorsed by the publisher.
